# Bats from different foraging guilds prey upon the pine processionary moth

**DOI:** 10.7717/peerj.7169

**Published:** 2019-07-05

**Authors:** Inazio Garin, Joxerra Aihartza, Urtzi Goiti, Aitor Arrizabalaga-Escudero, Jesús Nogueras, Carlos Ibáñez

**Affiliations:** 1 Zoologia eta Animali Zelulen Biologia Saila, UPV/EHU, Leioa, The Basque Country; 2 Evolutionary Ecology Department, Estación Biológica de Doñana, CSIC, Seville, Spain

**Keywords:** Moth pest, Pine forest, Bat ensemble, Foraging guild, Faecal DNA

## Abstract

Outbreaks of the processionary moth *Thaumetopoea pityocampa* (Denis & Schiffermüller, 1775), a forest pest from the Palearctic, are thought to induce a behavioral response of bats, but up to now the moth has been seldom identified as bats’ prey. Studies on bat diets suggest moths with cyclical outbreaks attract a wide array of bat species from different foraging guilds. We test whether bats feed upon *T. pityocampa* in the Iberian Peninsula irrespective of the predator’s ecological and morphological features. We found that seven out of ten bat species belonging to different foraging guilds contained *T. pityocampa* DNA in their faeces and no difference was found in the foraging frequency among foraging guilds. A different size of the typical prey or the lack of fondness for moths can explain the absence of the pest in some bat species. Moreover, the intraspecific foraging frequency of *T. pityocampa* also changed with the sampling site likely representing differential availability of the moth. Lack of information on flight and dispersal behavior or the tympanate nature of the adult moth complicates understanding how different foraging guilds of bats prey upon the same prey. Our data suggests that *T. pityocampa* is a remarkable food source for many thousands of individual bats in the study area and we anticipate that more bats besides the species studied here are consuming this moth.

## Introduction

The pine processionary moth *Thaumetopoea pityocampa* (Denis & Schiffermüller, 1775) is a defoliating forest pest affecting coniferous trees in the Western Palearctic. In addition to its effect on tree growth (Jactel et al., 2006; Kanat, Alma & Sivrikaya, 2005), the urticant setae of the larvae also are a health hazard for humans and animals (Moneo et al., 2015). Current control methods (2009/128/EC) largely involve direct application of a preparation containing spores and toxins from the bacterium *Bacillus thuringiensis kurstaki*, which disrupts midgut epithelial cells of arthropod larvae, with limited consequences on non-target lepidopterans (Rastall et al., 2003; Boulton et al., 2007). While this treatment has short term negative effects on the pest populations, the control treatment does not seem to suppress cyclical outbreaks, promoting a debate over the suitability of spraying as an efficient management action (Cayuela, Hódar & Zamora, 2011).

*T. pityocampa* also have a diverse array of natural predators, however, many of them are either occasional or incidental (Hódar, Torres-Muros & Senhadji, 2013) and mortality caused by most does not apparently affect population dynamics (Battisti et al., 2015). Bats and nightjars (*Caprimulgus* spp) are the main vertebrate consumers of the adult moth (Auger-Rozenberg et al., 2015), but because bats are overwhelmingly in higher numbers than nightjars, the former are expected to pose the main predatory threat to the moth.

Bats’ response to insect prey outbreaks is well known (Fukui et al., 2006; Mata et al., 2016; Krauel et al., 2017) and the array of moth species that bats eat include many damaging pests of crops. Moths detrimental to cotton, rice or corn are consumed readily by bats (McCracken et al., 2012; Puig-Montserrat et al., 2015; Maine & Boyles, 2015) and damage to crops increases where hunting by bats is precluded (Maas et al., 2016; Boyles et al., 2013). Further, a substantial part of the bat community is able to incorporate suddenly available prey into their diet (Russo, Bosso & Ancillotto, 2018). Empirical evidence to unequivocally sustain such a claim remains to be collected, but ancillary observations on moths with cyclical abundances (such as the migratory *Autographa gamma*, *Apamea monoglypha* or *Noctua pronuba*) suggest that they are eaten by very different bats in terms of flight capability, echolocation pattern, ecological habits like roost behavior or hunting grounds (Razgour et al., 2011; Zeale et al., 2011; Alberdi et al., 2012; Hope et al., 2014; Arrizabalaga-Escudero et al., 2015; Arrizabalaga-Escudero et al., 2018; Aizpurua et al., 2018).

The periodic outbreaks of *T. pityocampa* across Southwestern Europe make this organism a good model to describe the consumption of this pest by a rich bat ensemble. Bats have been already observed to respond to moth numbers by increasing the hunting activity along edges of infected forest stands (Charbonnier, Barbaro & Theillout, 2014). The only two bats species known to predate on *T. pityocampa* (*Rhinolophus euryale*, Arrizabalaga-Escudero, 2016; *Miniopterus schreibersii*, Aizpurua et al., 2018; Galan et al., 2018) belong to different foraging guilds (Denzinger & Schnitzler, 2013), catching prey at habitats that require different manoeuverability and flight speed (Goiti et al., 2006; Vincent, Nemoz & Aulagnier, 2011). Wing morphology of *T. pityocampa* suggests it flies relatively fast with reduced manoeuverability, what may increase its vulnerability to bats (Rydell & Lancaster, 2000; Jantzen & Eisner, 2008). On the other hand, its tendency to aggregate around forests’ edge areas to mate or lay eggs (Démolin, 1969) might expose it to bats adapted to fly both within and against clutter. Thus, we hypothesize that predation upon *T. pityocampa* is irrespective of the foraging characteristics of bats. Specifically, we expect the presence of *T. pityocampa* in the diet of bats from different foraging guilds. Thus, we analyzed the relationship between the consumption of *T. pityocampa* and the foraging guilds of bats using DNA metabarcoding.

## Materials & Methods

**Figure 1:**
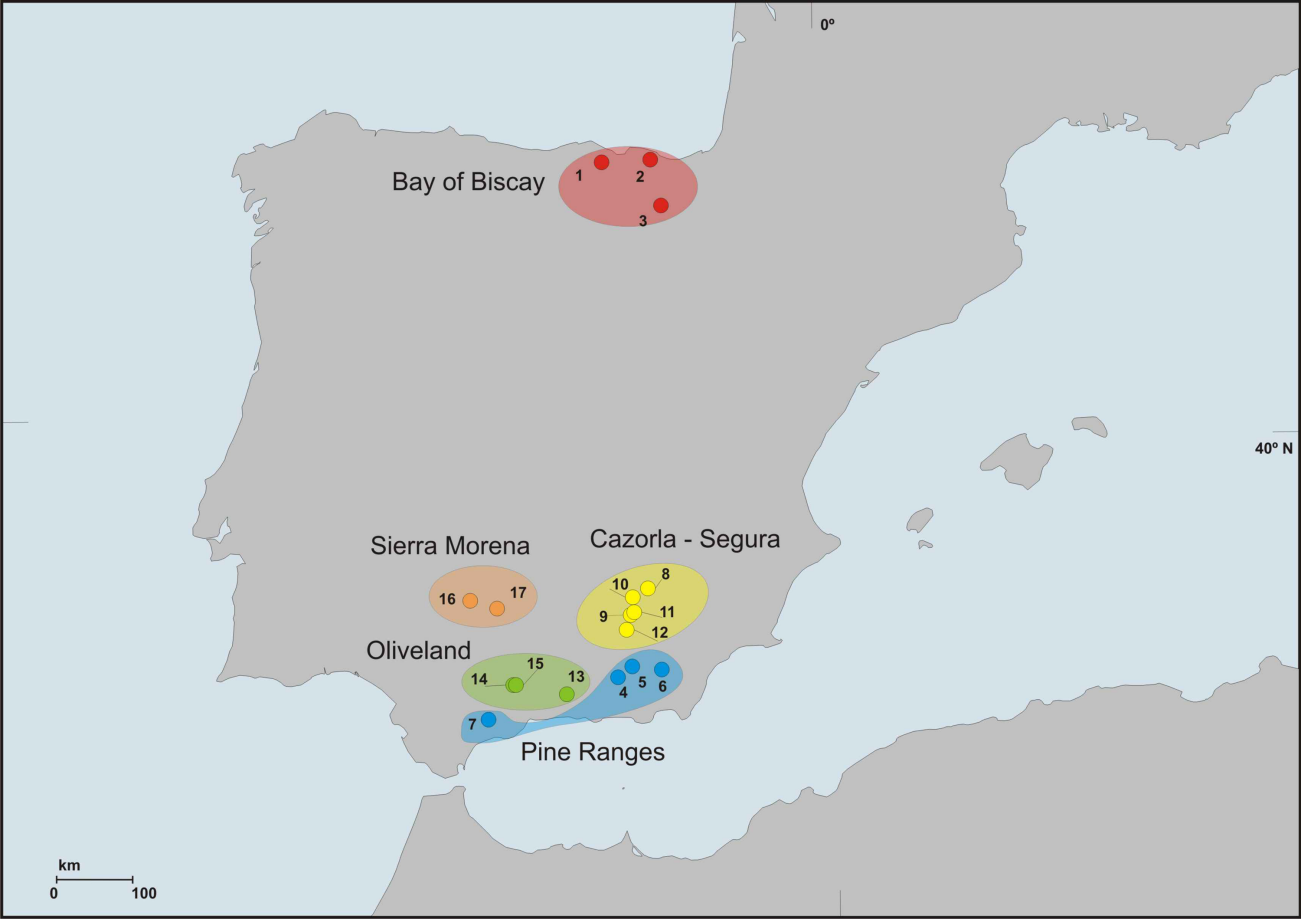
Geographic distribution of sampling locations.

We sampled bats at 17 locations from the Iberian Peninsula, three of them (Bay of Biscay) during July 2012 and the rest in July 2014 (Fig. 1). Bats were captured either with a 2 × 2 m harp trap (Tuttle, 1974) located at the entrance of the colony roosts from 00.30 a.m. onwards, as bats returned to them, or with mist nets set on ponds during sunset, aimed at drinking bats whose roosts were unknown. Captures at each location were conducted in a single night in order to minimize disturbance. Each bat was held individually in a clean cloth bag until it defecated (for a maximum of 40 min). Bats were identified to the species level, sexed and aged, their weight and forearm length were measured and their faecal material was collected. Faeces were frozen within 6 h from the moment of collection. After handling bats were immediately released at the capture site.

 All applicable international, national, and/or institutional guidelines for the care and use of animals were followed. Capture and handling protocols were approved by the Ethics Committee at the University of the Basque Country (Ref. CEBA/219/2012/GARIN ATORRASAGASTI). Captures were performed under license from the corresponding Regional Government’s body.

The following institutions issued permits for the capture and handling of bats: the Department of the Environment of the Regional Council of Araba (permit number 12/267); the Department of the Environment of the Regional Council of Biscay (G13 1061; G13 1064 and G13 1066); and the Department of the Environment of the Government of Andalusia (191/0400).

We classified the faecal sample according to the bat species, the bat foraging guild and the region. Classification of foraging guilds of bats followed Denzinger & Schnitzler (2013): open space aerial foragers or high flyers (*Tadarida teniotis* and *Nyctalus lasiopterus*), edge space aerial foragers or on-canopy hawkers (*Nyctalus leisleri*, *Hypsugo savii*, *Miniopterus schreibersii* and *Barbastella barbastellus*) and within canopy or narrow space foragers (*Rhinolophus* spp. and *Plecotus austriacus*). The studied bats encompass both moth specialists, such as *T. teniotis, M. schreibersii*, *B. barbastellus, Rhinolophus euryale* and *Plecotus austriacus*, and more generalist foragers, e.g., *N. lasiopterus, N. leisleri*, *H. savii*, *R. ferrumequinum* and *R. hipposideros*.

We grouped sampling locations into different *regions* (Fig. 1, Table 1) according to climatic, topographic and forest coverage features. The three locations from Bay of Biscay were wetter (annual rainfall above 900 mm) and colder (annual mean T < 12.5 °C) than the rest (330–780 mm; 12–17 °C). Altitudes at locations from Bay of Biscay ranged 80–845 m a.s.l.; at Segura–Cazorla altitudes were above 1,000 m; Pine Ranges reached >1,500 m; Sierra Morena 300–500 m; Oliveland 400–800 m. All location were moderately to highly forested except locations in Oliveland, which lacked woodland in the surroundings. The cover of conifer stands around locations showed the lowest values at Oliveland sites (2–4% of surface area), intermediates at Sierra Morena (12–59%), Pine Ranges (18–40%) and Bay of Biscay (2–60%), and the highest in Segura–Cazorla (30–89%). Most of the coniferous trees in the study locations (>75%) were pines (*Pinus* spp.).

**Table 1:** Number of bat individuals whose faeces were positive for *T. pityocampa* (numerator) and number of bats sampled (denominator). Bat species abbreviations are as follows: Bba, *Barbastella barbastellus* (Schreber, 1774); Hsa, *Hypsugo savii* (Bonaparte, 1837); Msc, *Miniopterus schreibersii* (Kuhl, 1817); Nla, *Nyctalus lasiopterus* (Schreber, 1780); Nle, *Nyctalus leisleri* (Kuhl, 1817); Pas, *Plecotus austriacus* (J.B. Fischer, 1829); Reu, *Rhinolophus euryale* Blasius, 1853; Rfe, *Rhinolophus ferrumequinum* (Schreber, 1774); Rhi, *Rhinolophus hipposideros* (Bechstein, 1800); Tte, *Tadarida teniotis* (Rafinesque, 1814).

**Regions**	**Narrow Space**				**Edge Space**				**Open Space**	
(no of locations)	Pas	Reu	Rfe	Rhi	Bba	Hsa	Msc	Nle	Nla	Tte
Bay of Biscay (3)		**0**/42	**4**/16	**0**/33			**2**/37			
Pine Ranges (4)	**23**/28	**23**/23				**0**/22	**2**/5	**0**/5		**5**/10
Cazorla-Segura (5)	**8**/16	**4**/8	**0**/5		**5**/7	**0**/8	**24**/40		**3**/5	
Oliveland (3)		**0**/13	**0**/35				**0**/19			
Sierra Morena (2)		**0**/22	**0**/22				**0**/21			

The defoliation level of the tree crown has been below 25% in more than 90% of the pines monitored yearly since 1990 (Sánchez-Peña et al., 2010). Incidence of defoliation caused by the pest varied locally with no clear-cut pattern among regions and, in general, the stands with the highest defoliation level (above 60%) at any location occurred farther than 10 km from the sampling location (own data and the Environmental Agency of the Government of Andalusia). Thus, a pest population can be confirmed in the study area, but we lacked any data on the abundance of moths during the sampling period across regions, either because their monitoring was no longer carried out or because the data were not available. Furthermore, some faecal samples were obtained more than 10 km far from the nearest pine stands, precluding the search for any relationship between the moth availability and consumption by bats.

We extracted DNA from faecal samples using the QIAamp DNA Stool Mini Kit (Qiagen, UK), following Zeale et al. (2011). An extraction blank –control– of each extraction series confirmed there was no DNA contamination. A 157 bp-long fragment of the mitochondrial cytochrome *c* oxidase subunit I barcode region (COI) was PCR-amplified from each DNA extract using modified ZBJ-ArtF1c and ZBJ-ArtR2c primers, which are successful in the amplification of DNA of moths (Zeale et al., 2011). Each modified primer consisted of the original ZBJ-ArtF1/R2c primer extended at the 5′ end by 10 bp Multiplex Identifiers (MIDs) and Ion Torrent adaptor sequences (Clare et al., 2014). Each sample was tagged with a unique combination of MID primers following Brown et al. (2014). These unique tags allowed the separation of each individual bat sample bioinformatically. PCR protocols were conducted primarily following Bohmann et al. (2011). We completed PCRs in a 20 µL reaction that contained 10 µL of Qiagen multiplex PCR (Qiagen CA) master mix, 6 µL of water, 1 µL of each 10 µM primer and 2 µl of DNA. Thermocycler conditions were: 95 °C for 15 min; 50 cycles of 95 °C –30 s, 52 °C –30 s, 72 °C –30 s; 72 °C –10 min. A negative PCR control added at each PCR series proved no crossover contamination. Each product was visualized on a 2% agarose pre-cast 96 well E-gel (Invitrogen, Life Technologies). Product size selection was performed using the PCRClean DX kit (Aline Biosciences). We eluted the product in water and measured the concentration on the Qubit 2.0 spectrophotometer using a dsDNA HS Assay Kit (Invitrogen, Life Technologies). We normalized the products to 1 ng/µL prior to final library dilution. Sequencing was conducted on the Ion Torrent (Life Technologies) sequencing platform using a 318 chip and following the manufacturers guidelines but using a 2x dilution.

We performed the bioinformatic analysis of obtained sequences in three main stages: (i) quality control, sequence pre-processing, and collapsing of identical sequences into unique sequences and singleton removal were performed using PRINSEQ 0.20.4 (Schmieder & Edwards, 2011), FASTX-Toolkit 0.0.13 (http://hannonlab.cshl.edu/fastx_toolkit/index.html) and AdapterRemoval (Lindgreen, 2012); (ii) clustering of sequences represented by more than one read into Molecular Operational Taxonomic Units (MOTU) was carried out with the QIIME *pick_otu* and *uclust* algorithms (Caporaso et al., 2010), and the screening for chimeric sequences was conducted using *chimera.uchime* command (UCHIME program; Edgar et al., 2011) by screening the reference sequences from each MOTU against >500,000 COI sequences representing arthropods download from Genbank (NCBInr/nt); (iii) after comparing a representative sequence of each MOTU against reference sequences in the Barcode Of Life Database (BOLD; http://www.boldsystems.org/) using the BLAST algorithm (Altschul et al., 1990), only MOTUs identified as *T. pityocampa* with a confidence higher than 98% (Clare et al., 2014) were considered.

We studied the effect of the bat foraging guild on the proportion of conspecific individuals from the same location that consumed the pest using the *glm* function in R (R Core Team, 2013) to perform a factorial analysis (Crawley, 2013). Although a geographical analysis of the pest consumption was not our study aim, we incorporated the variable region into the model as an additional explanatory variable because the origin of the sample (landscape configuration) could also play a role on the observed proportions. Unfortunately, we did not test the most complex model including the interaction term between foraging guild and region, as the high number of missing values of the high-flyer class in some regions prevented it. We corrected for overdispersion using a quasibinomial procedure as the preliminary modelization done with binomial error models showed overdispersion of residuals (Residual Deviance was four times the Residual degrees of freedom).

## Results

Seven out of the ten bat species consumed *T. pityocampa* at least at some location. Only *H. savii*, *N. leisleri* and *R. hipposideros* did not show any trace of the pest in their faeces (Table 1).

We did not find *T. pityocampa* in the faeces of any bat from Sierra Morena and Oliveland, although individuals from the same bat species were positive in other locations. Among the positive locations and positive bats, we found the highest consumption frequencies at the Pine Ranges region (80% of individuals) and *P. austriacus* species (70%). *M. schreibersii* (34%) consumed the moth in five out of nine of the studied locations, whereas *R. euryale* (36%) and *R. ferrumequinum* (5%) only did in two out of seven and six locations respectively. At least half of *B. barbastellus*, *N. lasiopterus* and *T. teniotis* individuals fed upon *T. pityocampa*, although their sample size was limited, both in terms of number of individuals and locations. In some locations where *M. schreibersii*, *R. euryale* and *R. ferrumequinum* were syntopic, one (or two) species consumed the moth whereas the remainder did not.

The simplest generalized linear model (*Residual deviance* = 127, *d.f.* = 29) fitted to the data kept Region as the only significant variable affecting the proportion of bats consuming the pest (*Deviance* = 179, *d.f*. = 4, *Residual d.f.* = 29, *p* < 0.001). It was not significantly different from the model containing Foraging guild and Region as explanatory variables (*Difference in Deviance* = −20.2, *F* = 2.96, *d.f.* = 2, *p* = 0.07).

## Discussion

More than half of the scrutinized bat species consumed *T. pityocampa*, regardless of the foraging guild of the bat. The results indicate that, in general terms, the bat ensemble is responsive to this pest. Besides the two species previously noted as preying upon it, namely *R. euryale* and *M. schreibersii* (Arrizabalaga-Escudero, 2016; Aizpurua et al., 2018; Galan et al., 2018) our study has unfolded predation by another five. Remarkably, we found that *T. pityocampa* is also eaten by *R. ferrumequinum*, a horseshoe bat previously discarded as a potential consumer of this pest because it has seldom been reported using pine stands as hunting grounds (Auger-Rozenberg et al., 2015). *P. austriacus* and *B. barbastellus* had been already appointed as potential predators of *T. pityocampa* (Charbonnier, Barbaro & Theillout, 2014; Auger-Rozenberg et al., 2015) whereas *T. teniotis* and *N. lasiopterus* have never been referred to. The low dependency of *H. savii* on moths (Beck, 1995; Horáček & Benda, 2004; Whitaker & Karataş, 2009) may explain the differences observed between this and other edge space foragers. Further, we cannot rule out that *T. pityocampa* displays evasive flight when hearing bat calls emitted at frequencies between 20 and 50 kHz (Surlykke, 1984), so that its availability as prey for species such as *H. savii* or *N. leisleri* would be effectively reduced (Waters, 2003). This medium-sized moth may be out of the reachable prey size range of the smallest of the studied bats, *R. hipposideros*, a within-forest narrow-space hunter that usually consumes tiny prey even when feeding upon moths (Andreas et al., 2013; Galan et al., 2018). Beyond the aforementioned constraints, the processionary moth does not show any morphological feature that requires particular predatory traits during handling, biting or chewing and in that respect we suspect all studied bats as well as other European ones may perform similarly during the acquisition of this pest species. We expect bats that prey upon moths in a regular or occasional basis in Southern Europe, that are large enough to handle medium-sized moths as *T. pityocampa*, that echolocate below or above the alleged hearing frequency range of notodontid moths, and are susceptible to encounter it within their typical commuting range, are also consuming the pest, namely *Plecotus auritus*, *Rhinolophus mehelyi*, *R. blasii*, or *Nyctalus noctula*. If we were right, they would add an impressive task force into the community of predators that can fight this pest moth’s outbreaks.

We found bats from every foraging guild consumed *T. pityocampa*, concurrently disproving any dependence between the bat guild and the presence of the pest in the diet of bats. Similarly, the hemipteran pest *Nezara viridula* has been reported as prey of several bats belonging to the same three foraging guilds in South Africa (Taylor et al., 2017). Dynamic changes in wing conformation, wing mass distribution and mechanical features of wing tissues make bats versatile flyers (Swartz, Freeman & Stockwell, 2003) allowing them to exploit a broad section of the aerospace. Female moths fly two to three times during their apparently short lifespan after emergence and the average travel distance in search of the right twig to lay eggs is 2–5 km, occasionally over open ground (Démolin, 1969; Battisti et al., 2015). Less is known about the behavior of males in search of mates, although experiments with captive individuals revealed they are able to fly around 20 km on average (Battisti et al., 2015). Provided the moths fly over open ground (Démolin, 1969), they may become readily available for the open space flying bats. Hitherto there is, however, no empirical evidence supporting such behaviour by *T. pityocampa*. Activity of both sexes appears to be higher at the forest edge and canopy level (Démolin, 1969; Jactel et al., 2006). Likely, the fondness of the pest for twigs at the tree edge (both to mate and for oviposition) makes them to aggregate in vast numbers in the interface, where the hunting space of bats with contrasting flight morphology meets. In turn, bats can easily foresee the moth’s location once first imagines have emerged. Foraging by different bat guilds would synergistically suppress this pest’s populations, with small narrow–space bats hunting upon the moth at the stand level and the high flyers preventing infestation of new stands.

*T. pityocampa* was not present in the diet of all bat species from every guild neither in all individuals from the same species and location. We cannot discard that the intraspecific differences observed between locations may be related to a contrasting availability of the pest due to the local timing of adult emergence. Zhang & Paiva (1998), using pheromone traps, reported a major peak of seasonal flight in early September at several locations of the Iberian Peninsula with a trend to fly earlier at higher altitudes. Thus, future detection of similar pests in bats‘ diet will require a thorough seasonal survey. Nevertheless, the observed consumption pattern by some bats cannot only be explained by differential availability of the pest. For example, the greater horseshoe bat (*R. ferrumequinum*) did prey on the pest in some places where the Mediterranean horseshoe bat (*R. euryale*) did not and vice versa. The observations on these two bats suggest that absolute availability of the pest is not the only variable determining consumption and maybe its relative profitability accounts for it too.

## Conclusions

The high mobility and large lifespan of bats are features that likely promote their ability to capitalize on the transient nature of pest outbreaks (Boyles et al., 2013) and thus render also this group a putative biocontrol agent, at least ideally. Although bats in general have been previously quoted as predators of *T. pityocampa* (Charbonnier, Barbaro & Theillout, 2014; Roques, 2015), we offer for the first time unambiguous evidence on predation by a significant number of bat species from different foraging guilds over a wide geographical scale. Lately, a number of studies have revealed the so far overlooked role of bat predation in crop damage reduction (reviewed in Maas et al., 2016). Unfortunately, our study cannot shed more light on the capability of bats to numerically control *T. pityocampa* and reduce damage because we could only assess presence of DNA from *T. pityocampa* in bat faeces and were not able to estimate prey number or biomass consumed per individual. Even though, if we conservatively accept the minimal predation rate on the pest by each bat individual (one moth per bat and foraging bout), extrapolation for the entire bat population (>240,000 individuals of *M. schreibersii*, >70,000 *P. austriacus*, 38,000 *R. euryale*, or >45,000 *R. ferrumequinum* estimated in the Iberian Peninsula; (Palmeirim & Rodrigues, 1992; Palomo, Gisbert & Blanco, 2007) and the flight period of the moth results in figures to be not neglected. Nevertheless, any precise picture of the bats consuming the pest at regional or local scale requires at least (1) a previous survey of the active period of the imago phase at the study site, (2) a general knowledge on the factors affecting its availability across the landscape and (3) to include as many foraging guilds as possible among the monitored bats. Further, the current knowledge on the dispersal biology of the adult stage of *T. pityocampa* is poor, what precludes the establishment of the precise basis of the interaction between the pest and bats. As a result, it does not only reduce our ability to manage bat populations as putative control agents but it also might call into question whether they play any effective role in the biological control of this pest.

##  Supplemental Information

10.7717/peerj.7169/supp-1 File S1 Nucleotide sequences of the MOTUs used to assign the identity of the pine processionary moth Bats species and the region where the MOTUs assigned to *Thaumetopoea pityocampa* were observed as well as the number of sequences comprising the MOTU and the confidence % of the taxonomic assignation are indicated. Species abbreviations follow Table 1.

## Additional Information and Declarations

### Field Study Permissions

The following information was supplied relating to field study approvals (i.e., approving body and any reference numbers): The following institutions issued permits for the capture and handling of bats: Department of the Environment of the Regional Council of Araba (Permit number 12/267); Department of the Environment of the Regional Council of Biscay (G13 1061; G13 1064 and G13 1066); and Department of the Environment of the Government of Andalusia (191/0400).

### Data Availability

The following information was supplied regarding data availability: Nucleotide sequences of the MOTUs used to assign the identity of the pine processionary moth are available in the Supplemental File.
