# Supplementary material for: Bats from different foraging guilds prey upon the pine processionary moth
Source: PeerJ. 2019 Jul 5;7:e7169. doi: 10.7717/peerj.7169 (PMC6613530; doi:10.7717/peerj.7169)
Supplement: File S1 — Bats species and the region where the MOTUs assigned to Thaumetopoea pityocampa were observed as well as the number of sequences comprising the MOTU and the confidence % of the taxonomic assignation are indicated. Species abbreviations follow Table 1. [file peerj-07-7169-s001.docx]

MOTUs assigned to *Thaumetopoea pityocampa*. Bats species and the region where the MOTU was observed as well as the number of sequences comprising the MOTU and the confidence % of the taxonomic assignation are indicated. See Table 1 for bat species abbreviations.

| **Bat sp.** | **No of Seq.** | **Region (no individuals)** | **Confidence Percentage** | **MOTU** |
| --- | --- | --- | --- | --- |
| Bba | 13 | Cazorla-Segura (5) | 100 | AATTTGGGCAGGAATAGTAGGCACATCATTAAGATTACTAATTCGAGCAGAATTAGGAACCCCCGGATCTTTAATTGGTGATGACCAAATCTATAACACAATTGTCACAGCCCATGCTTTTATTATAATTTTTTTCATGGTAATACCAATTATAATT |
| Nla | 21 | Cazorla-Segura (3) | 100 | AATTTGGGCAGGAATAGTAGGCACATCATTAAGATTACTAATTCGAGCAGAATTAGGAACCCCCGGATCTTTAATTGGTGATGACCAAATCTATAACACAATTGTCACAGCCCATGCTTTTATTATAATTTTTTTCATGGTAATACCAATTATAATT |
| Msc | 5 | Cazorla-Segura (3) | 100 | AATTTGAGCTGGAATAGTTGGAACTTCATTAAGATTACTAATTCGAGCAGAATTAGGAACCCCCGGATCTTTAATTGGTGATGACCAAATCTATAACACAATTGTCACAGCCCATGCTTTTATTATAATTTTTTTCATGGTAATACCAATTATAATT |
| Msc | 906 | Pine Ranges (2); Cazorla-Segura (24); Bay of Biscay (2) | 100 | ATTTGGGCAGGAATAGTAGGCACATCATTAAGATTACTAATTCGAGCAGAATTAGGAACCCCCGGATCTTTAATTGGTGATGACCAAATCTATAACACAATTGTCACAGCCCATGCTTTTATTATAATTTTTTTCATGGTAATACCAATTATAATT |
| Msc | 1 | Pine Ranges (1) | 99.21 | GGCTTGAGCTGGAATAGTTGGAACTTCTTTAAGTTTACTAATTCGAGCAGAATTAGGAACCCCCGGATCTTTAATTGGTGATGACCAAATCTATAACACAATTGTCACAGCCCATGCTTTTATTATAATTTTTTTCATGGTAATACCAATTATAATT |
| Msc | 3 | Cazorla-Segura (1) | 99.21 | TATTTGAGCAGGTATAATTGGAACTTCTTTAAGATTACTAATTCGAGCAGAATTAGGAACCCCCGGATCTTTAATTGGTGATGACCAAATCTATAACACAATTGTCACAGCCCATGCTTTTATTATAATTTTTTTTCATGGTAATACCAATTATAATT |
| Msc | 18 | Cazorla-Segura (4) | 99.21 | AATTTGATCAGGAATAGTAGGAACTTCTTTAAGATTATTAATTCGAGCAGAATTAGGAACCCCCGGATCTTTAATTGGTGATGACCAAATCTATAACACAATTGTCACAGCCCATGCTTTTATTATAATTTTTTTCATGGTAATACCAATTATAATT |
| Pas | 1 | Cazorla-Segura (1) | 100 | AGCCTGAGCTGGAATAGTAGGCACATCATTAAGATTACTAATTCGAGCAGAATTAGGAACCCCCGGATCTTTAATTGGTGATGACCAAATCTATAACACAATTGTCACAGCCCATGCTTTTATTATAATTTTTTTCATGGTAATACCAATTATAATT |
| Pas | 1943 | Pine Ranges (23); Cazorla-Segura (8) | 100 | ATTTGGGCAGGAATAGTAGGCACATCATTAAGATTACTAATTCGAGCAGAATTAGGAACCCCCGGATCTTTAATTGGTGATGACCAAATCTATAACACAATTGTCACAGCCCATGCTTTTATTATAATTTTTTTCATGGTAATACCAATTATAATT |
| Pas | 2 | Pine Ranges (1) | 98.41 | AATTTGAGCAGGAATAGTAGGAACATCTTTAAGTTTACTAATTCGAGCTGAATTAGGAACCCCCGGATCTTTAATTGGTGATGACCAAATCTATAACACAATTGTCACAGCCCATGCTTTTATTATAATTTTTTTCATGGTAATACCAATTATAATT |
| Pas | 3 | Cazorla-Segura (1) | 98.41 | GATTTGAGCAGGTATGGTAGGAACTTCCTTAAGATTGTTAATTCGAGCAGAATTAGGAACCCCCGGATCTTTAATTGGTGATGACCAAATCTATAACACAATTGTCACAGCCCATGCTTTTATTATAATTTTTTTCATGGTAATACCAATTATAATT |
| Pas | 4 | Cazorla-Segura (1) | 98.06 | AATTTGGGCAGGAATAGTAGGCACATCATTAAGATTACTAATTCGAGCAGAATTAGGAACCCCGGATCTTTAACTTGGTGATGACCAAATCTATAACACAATTGTCACAGCCCATGCTTTTATTATAATTTTTTTTATAGTTATACCTATTATAATT |
| Pas | 50 | Pine Ranges (10); Cazorla-Segura (3) | 100 | AATTTGGGCAGGAATAGTAGGCACATCATTAAGATTACTAATTCGAGCAGAATTAGGAACCCCCGGATCTTTAATTGGTGATGACCAAATCTATAACACAATTGTCACAGCCCATGCTTTTATTATAATTTTTTCATGGTAATTACCAATTATAATT |
| Pas | 12 | Pine Ranges(2) | 99.21 | TATCTGAGCTGGGATAGTAGGAACTTCATTAAGATTACTAATTCGAGCAGAATTAGGAACCCCCGGATCTTTAATTGGTGATGACCAAATCTATAACACAATTGTCACAGCCCATGCTTTTATTATAATTTTTTTTCATGGTAATACCAATTATAATT |
| Pas | 1 | Cazorla-Segura (1) | 98.04 | AATTGGGCAGGAATAGTAGGCACATCATTAAGATTACTAATTCGAGCAGAATTAGGAACCCCCGGATCTTTAATTGGTGATGACCAAATCTATAACACAATTGTCACAGCCCATGCTTTTATTATAATTTTCTTTATAGTAATACCAATTGTAATT |
| Reu | 2 | Pine Ranges (1) | 100 | AATTTGAGCTGGTATAGTAGGAACTTCATTAAGATTACTAATTCGAGCAGAATTAGGAACCCCCGGATCTTTAATTGGTGATGACCAAATCTATAACACAATTGTCACAGCCCATGCTTTTATTATAATTTTTTTTCATGGTAATACCAATTATAATT |
| Reu | 5 | Pine Ranges (3) | 100 | TGGGATGCAGGAATAGTAGGCACATCATTAAGATTACTAATTCGAGCAGAATTAGGAACCCCCGGATCTTTAATTGGTGATGACCAAATCTATAACACAATTGTCACAGCCCATGCTTTTATTATAATTTTTTTCATGGTAATACCAATTATAATT |
| Reu | 23 | Pine Ranges (15) | 100 | AATTTGGGCAGGAATAGTAGGCACATCATTAAGATTACTAATTCGAGCAGAATTAGGAACCCCGGATCTTTAACTTGGTGATGACCAAATCTATAACACAATTGTCACAGCCCATGCTTTTATTATAATTTTTTTCATGGTAATACCAATTATAATT |
| Reu | 1572 | Pine Ranges (22); Cazorla-Segura (4) | 100 | ATTTGGGCAGGAATAGTAGGCACATCATTAAGATTACTAATTCGAGCAGAATTAGGAACCCCCGGATCTTTAATTGGTGATGACCAAATCTATAACACAATTGTCACAGCCCATGCTTTTATTATAATTTTTTTCATGGTAATACCAATTATAATT |
| Rfe | 7 | Bay of Biscay (4) | 100 | AATTTGGGCAGGAATAGTAGGCACATCATTAAGATTACTAATTCGAGCAGAATTAGGAACCCCCGGATCTTTAATTGGTGATGACCAAATCTATAACACAATTGTCACAGCCCATGCTTTTATTATAATTTTTTTCATGGTAATACCAATTATAATT |
| Tte | 4 | Pine Ranges (1) | 99.21 | AATTTGAGCAGGAATAGTTGGAACATCTTTAAGATTATTAATTCGAGCAGAATTAGGAACCCCCGGATCTTTAATTGGTGATGACCAAATCTATAACACAATTGTCACAGCCCATGCTTTTATTATAATTTTTTTTCATGGTAATACCAATTATAATT |
| Tte | 534 | Pine Ranges (5) | 100 | ATTTGGGCAGGAATAGTAGGCACATCATTAAGATTACTAATTCGAGCAGAATTAGGAACCCCCGGATCTTTAATTGGTGATGACCAAATCTATAACACAATTGTCACAGCCCATGCTTTTATTATAATTTTTTTCATGGTAATACCAATTATAATT |
